# Aegeline mitigates isoproterenol-induced myocardial infarction: insights from biochemical, histopathological, and in silico studies

**DOI:** 10.3389/fcvm.2026.1805096

**Published:** 2026-07-21

**Authors:** Sami I. Alzarea, Muhammad Afzal, Misbahuddin Rafeeq, Alaa Hamed Habib, Tariq G. Alsahli, Omar Awad Alsaidan, Hassan H. Alhassan, Abdulaziz Ibrahim Alzarea, Aseel Awad Alsaidan, Nadeem Sayyed, Imran Kazmi

**Affiliations:** 1 Department of Pharmacology, College of Pharmacy, Jouf University, Sakaka, Al-Jouf, Saudi Arabia; 2 Department of Pharmaceutical Sciences, Pharmacy Program, Batterjee Medical College, Jeddah, Saudi Arabia; 3 Division of Basic Medical Sciences, College of Medicine, Dhofar University, Salalah, Oman; 4 Department of Physiology, Faculty of Medicine, King Abdulaziz University, Jeddah, Saudi Arabia; 5 Department of Pharmaceutics, College of Pharmacy, Jouf University, Sakaka, Al-Jouf, Saudi Arabia; 6 Department of Clinical Laboratory Sciences, College of Applied Medical Sciences, Jouf University, Sakaka, Al-Jouf, Saudi Arabia; 7 Department of Clinical Pharmacy, College of Pharmacy, Jouf University, Sakaka, Al-Jouf, Saudi Arabia; 8 Department of Family and Community Medicine, College of Medicine, Jouf University, Sakaka, Saudi Arabia; 9 Department of Pharmacology, Dr. R. G. Bhoyar Institute of Pharmaceutical Education & Research, Wardha, Maharashtra, India; 10 Department of Biochemistry, Faculty of Sciences, King Abdulaziz University, Jeddah, Saudi Arabia

**Keywords:** aegeline, cardioprotective, isoproterenol, molecular docking, molecular dynamics simulation, myocardial infarction

## Abstract

**Background:**

Myocardial infarction (MI) remains a major cause of morbidity and mortality worldwide. Although considerable advances have been made in its diagnosis and management, the development of safe and effective cardioprotective agents remains an active area of investigation. Natural products continue to attract interest as potential therapeutic candidates because of their diverse pharmacological activities. Aegeline, an alkaloidal amide isolated from *Aegle marmelos*, has demonstrated antioxidant and anti-inflammatory properties; however, its cardioprotective effects in experimental myocardial injury have not been fully characterized.

**Objective:**

The aim of this work was to assess the cardioprotective effects of aegeline in an experimental MI model.

**Methods:**

Isoproterenol (ISO) (85 mg/kg, subcutaneouslySC) was employed to trigger acute MI in experimental rats. Aegeline was administered to the rats at doses of 5 and 10 mg (p.o.) on the 29th and 30th day, respectively, followed by ISO (85 mg/kg) (s.c.) on the same days.

**Results:**

Aegeline exhibited cardioprotective properties against ISO-induced damage, as evidenced by the amelioration of altered histopathological features, reduction in myocardial damage, and improvements in antioxidant and oxidative stress markers, inflammatory indicators, and signaling pathways, along with inhibition of apoptotic expression. In addition, aegeline did not affect liver or kidney function. Molecular docking using AutoDock Vina v1.2.6 revealed the strong binding affinity of aegeline for CK-MB (PDB ID: 3B6R), with key hydrophobic, hydrogen-bonding, and salt bridge interactions. Additional targets included LDH (7DBJ), cTnT (1J1D), and cTnI (7SC2), though CK-MB exhibited the most favorable interaction profile. Molecular dynamics simulations supported these findings through principal component analysis, dynamic cross-correlation, free energy landscape mapping, and generalized born surface area (MM-GBSA) binding free energy analysis.

**Conclusion:**

This study demonstrates that aegeline attenuates experimental myocardial injury by reducing oxidative stress, inflammation, and apoptosis. Computational analyses support potential molecular interactions with proteins associated with myocardial injury, while the biochemical and histopathological findings provide primary evidence for its cardioprotective activity.

## Introduction

Cardiovascular diseases (CVDs) remain the leading cause of death in humans and have emerged as a significant socioeconomic burden worldwide, with increasing prevalence (1). Myocardial infarction (MI) occurs mostly during the unstable phase of coronary atherosclerosis, when the arterial wall becomes swollen. Chronic ischemia results in irreversible cardiac damage, including extracellular matrix degradation and cardiomyocyte death (2). Despite significant advancements in patient care and lifestyle fluctuations, MI remains an imminent threat to global health, accounting for 15% of annual worldwide fatalities (3).

Therapeutic strategies based on natural products may provide a valuable source of safe, effective, and reasonably low-cost innovative treatments for MI. *Aegle marmelos* Linn., also known as bael, belongs to the Rutaceae family and has been widely used in indigenous Indian remedies due to its numerous therapeutic properties. Numerous compounds have been identified, with alkaloids and coumarin derivatives being the primary components (4). This plant offers various medicinal and therapeutic benefits, including anticancer (5), hypoglycemic (6, 7), anti-inflammatory and antipyretic (8), antimalarial (9), antimicrobial (10), antispermatogenic (10), antioxidant (11, 12), and ulcer-healing potential (13). It has previously been demonstrated that the alkaloidal amide aegeline exhibits antihyperglycemic and antidyslipidemic properties in verified animal models of type 2 diabetes (14). According to another study, aegeline increases glucose transport via parallel pathways regulated by Akt and Rac1, and displays cytoskeletal effects in skeletal muscle cells by stimulating the PI3-kinase-Rac1-PAK1-cofilin pathway. As a result, aegeline may act on multiple targets to improve insulin sensitivity in skeletal muscle cells (15). Furthermore, by reducing reactive oxygen species (ROS) production and increasing hydrogen sulfide, aegeline lessens the progression of increased glucose on kidney fibrosis (16). Aegeline reduces lipid abnormalities in hypercholesterolemic models more effectively than atorvastatin by activating oxidized low-density lipoprotein receptor-1 (LOX-1), which resembles a lectin (17). Aegeline also prevents histamine from being released. Furthermore, aegeline acts as a histamine H1 receptor antagonist by interacting with amino acid residues Asp107, Lys179, Lys191, Asn198, and Trp428 of the receptor (18).

The experimental MI model centered on isoproterenol (ISO) is widely used in research because it mimics MI-induced damage in humans (19). ISO-induced myocardial injury is caused by a range of mechanisms, including increased ROS production, cytokine activation, altered cell signaling pathways, and mitochondrial damage, all of which ultimately lead to cardiac failure. Therefore, treatment strategies aimed at reducing inflammation and oxidative stress may help prevent MI and other CVDs (20).

Although aegeline has been reported to possess antioxidant, anti-inflammatory, and antidiabetic properties, its protective effects against MI in the context of diabetes remain insufficiently characterized. Furthermore, limited information is available regarding the molecular mechanisms underlying its cardioprotective activity. Therefore, the present study was designed to investigate the effects of purified aegeline in an ISO-induced MI model under diabetic conditions using integrated biochemical, histopathological, and computational approaches. Using Autodock Vina V. 1.2.6, the docking study of compound interactions was performed on CK-MB (3B6R), LDH (7DBJ), cTnT (1J1D), and cTnI (7SC2). Binding energy, affinity, and interaction types at the active sites of the selected targets were analyzed to evaluate the docking outcomes. In addition, molecular dynamics (MD) simulations were conducted to assess the stability and convergence of 3B6R_Aegeline and 7DBJ_Aegeline.

## Materials and methods

### Chemicals

ISO and aegeline were obtained from MSW Pharma, M.S., India. All other consumables were of analytical grade and sourced from a certified supplier.

### Animals

Wistar experimental rats weighing 180 ± 20 g were used. Wistar rats were chosen for their well-defined physiology, ease of handling, and proven reliability in cardiovascular and pharmacological research. The research proposal (IAEC-PT-23-39) was approved by the Institutional Ethics Committee, and the ARRIVE guidelines were followed for conducting the research (21). The rats were kept in typical workroom conditions under accepted light–dark cycles. They were provided a standard pellet diet and had unlimited access to water. The experimental animals underwent a 1-week acclimatization period.

### Establishment of the rat MI model

To induce experimental MI, ISO (85 mg/kg, subcutaneously (s.c.) was dissolved in 1 mL of saline and administered to the animals at 24-h intervals for 2 days (29th and 30th day) (22).

### Treatment protocol

Following a 1-week acclimatization period, the experimental rats were randomly allocated into four groups (*n* = 6 per group), totaling 24 animals.

Group I (Control): Rats were given normal saline.

Group II (ISO): Rats were administered ISO (85 mg/kg, s.c.) on the 29th and 30th day.

Group III (ISO + Aegeline-5): Rats were given aegeline 5 mg per oral (p.o.) (23, 24) for 30 days + ISO (85 mg/kg) (s.c.), on the 29th and 30th day.

Group IV (ISO + Aegeline-10): Rats were given aegeline 10 mg per oral (p.o.) (23, 24) for 30 days + ISO (85 mg/kg) (s.c.), on the 29th and 30th day.

Doses were adjusted according to the rats’ body weights, which were recorded at both the initial and final stages. All animals were sacrificed 48 h following the initial ISO injection. Blood samples were collected from the retro-orbital sinus using heparinized capillary tubes. Centrifugation was employed to separate serum from the blood. At the end of the study, the experimental rats were anesthetized with ketamine (80 mg/kg, i.p.) and xylazine (10 mg/kg, i.p.), then sacrificed through cervical dislocation. The left ventricle myocardial tissues were carefully excised for assessment of biochemical parameters, including oxidative stress and antioxidant markers, as well as for histopathological examination of myocardial tissue changes across all experimental groups. Cardiac samples were separated and rinsed in ice-cold phosphate-buffered saline. A tissue homogenizer was then used to homogenize the samples in phosphate buffer solution (25 mM, pH 7.4), yielding approximately 10% w/v homogenate. After centrifuging the homogenate for 10 min at 1,700 rpm, the supernatant was collected and stored at −20°C for further biochemical analysis. In addition, 10% formalin was used to preserve some of the cardiac samples.

### Evaluation parameters

#### Body weight

A digital weighing balance was used to record the rats’ body weights.

#### Cardiac injury marker estimation

The levels of creatine kinase-MB (CK-MB) (25), lactate dehydrogenase (LDH) (26), cardiac troponin T (cTnT), and cardiac troponin I (cTnI) (27) were measured according to the supplier’s instructions. A solid-phase chemiluminescence enzyme immunoassay measured serum cortisol (28). Aldosterone was determined by a validated immunoassay. (29).

#### Antioxidants and oxidative and nitrosative stress markers

The tissue homogenate was centrifuged, and the supernatant was used for testing. Standard spectrophotometric methods were used to determine glutathione (GSH) (30), catalase (CAT) (31), and malondialdehyde (MDA) (32) levels for the assessment of antioxidant status and oxidative stress. ROS values were determined (33). Total nitrite/nitrate (NOx) concentrations were analyzed as per the manufacturer’s instructions (34).

#### Inflammatory and apoptotic caspase-3 activity

Enzyme-linked immunosorbent assay (ELISA) is a widely used technique to quantify proteins, such as inflammatory markers (IL-1β, TNF-α, IL-6, and NF-*κ*B), in rat tissue homogenate samples. The assay employed microwell plates precoated with specific antibodies. The target protein was bound to the capture antibody, followed by an enzyme-linked secondary antibody, forming a sandwich complex. Upon addition of a substrate, a colorimetric reaction occurred. Absorbance was measured at 450 nm using an ELISA reader. An ELISA kit was used to measure caspase-3 levels, and the results were expressed in ng/mL (35).

#### Hepatic and kidney function markers

Serum concentrations of hepatic enzymes—aspartate aminotransferase (AST) and alanine aminotransferase (ALT) (36)—as well as creatinine, and urea (37), were assessed by colorimetric analysis using commercially available kits according to the manufacturer’s instructions (Modern Lab., Maharashtra, India).

#### Histopathological architecture

Myocardial tissue samples were fixed in 10% buffered formalin, washed, and dehydrated using graded isopropyl alcohol. The dehydrated tissues were then embedded in molten paraffin wax and sectioned for histological evaluation. The sections were stained with hematoxylin and eosin (H&E) to assess structural alterations. Histopathological changes were examined under a light microscope (38). For quantitative histopathological assessment, myocardial sections were examined by a blinded pathologist using a semiquantitative injury scoring system. Cardiac injury was graded on a scale of 0–4 based on the severity of myocardial degeneration, edema, inflammatory cell infiltration, and tissue architecture disruption, where 0 = normal histology, 1 = mild injury, 2 = moderate injury, 3 = marked injury, and 4 = severe diffuse myocardial damage.

### Molecular docking

For the molecular docking study, the target protein X-ray crystallographic structures of CK-MB (3B6R), LDH (7DBJ), cTnT (1J1D), and cTnI (7SC2) were obtained from the Protein Data Bank at RCSB (https://www.rcsb.org/) (39). When selecting a set of proteins for docking studies, each PDB entry should be validated using X-ray crystallography, with a resolution below 2 Å. In addition, wwPDB validation should be performed, along with ensuring the presence of a native ligand and a Ramachandran plot. The target protein was refined using CHIMERA V1.16 (40) to ensure high-quality docking INPUT. Standard residues were minimized applying the AMBER force field, while non-standard residues were optimized with AM1-BCC semiempirical charges to preserve proper electrostatic interactions. Unnecessary elements, such as water molecules, cocrystal ligands, and redundant chains, were removed to avoid disruption during docking. The structure of aegeline was generated using Marvin Sketch, followed by hydrogen addition, 2D and 3D cleaning, and generation of possible conformers, which were selected and saved in mol2 format as the lowest-energy conformers. CHIMERA, with the AM1-BCC force field, was used to optimize the structure further. Then, the AutoDock tool was used to convert both proteins and ligands to PDBQT format (41), which is compatible with the Vina tool, with ligand rotatable bonds set flexible to allow conformational changes and proteins kept rigid (42). Docking simulations were performed without specific solvent requirements. Active site amino acid residues were identified for each target by analyzing the presence of cocrystallized ligands and CASTp (https://sts.bioe.uic.edu/castp/index.html) (43) for apo structures. Based on the identified active sites (Table 1), a grid box was established, with its dimensions adjusted to include the region where the protein ligand interacts. The native ligand was surrounded by grid centers, as detailed in Table 2.

**Table 1:** Active-site amino acid residues identified in molecular docking studies of aegeline with proteins associated with myocardial injury.

Target	PDB ID	Active site amino acids
CK-MB	3B6R	THR59B, ASN63B, GLY65B, HIS66B, ILE69B, MET70B, THR71B, VAL72B, GLY73B, CYS74B, ARG96B, SER128B, ARG130B, ARG132B, ILE188B, ASP190B, HIS191B, LEU193B, PHE194B, ASP195B, LYS196B, SER199B, PRO200B, LEU201B, LEU202B, TRP228B, GLU231B, GLU232B, ARG236B, MET240B, LYS242B, CYS283B, SER285B, ASN286B, ARG292B, GLY294B, VAL295B, HIS296B, GLN318B, ARG320B, THR322B, GLY323B, GLY324B, VAL325B, ASP326B, ASP335B
LDH	7DBJ	SER203A, GLY204A, ASN206A, GLY209A, LYS308A, GLY204C, ASN206C, GLY209C, SER211C, LYS308C, LYS310C
cTnT	1J1D	GLU94D, GLU95D, SER98D, ASP99D, TYR150D, TYR259E, GLU260E, VAL263E, LEU264E, ARG267E, ILE125F, THR129F
cTnI	7SC2	PHE27A, ILE36A, LEU41A, MET45A, MET60A, VAL64A, VAL72A, PHE77A, MET80A

**Table 2:** Grid box parameters used for molecular docking of aegeline with proteins associated with myocardial injury.

Target	PDB ID	Center-coordinates			Size-coordinates		
		*x*	*y*	*z*	*x*	*y*	*z*
CK-MB	3B6R	−54.6552	6.1184	9.8609	20	20	20
LDH	7DBJ	15.42	−0.47	−29.36	20	20	20
cTnT	1J1D	23.8969	105.26	23.3956	20	20	20
cTnI	7SC2	2.6239	15.6685	12.0271	20	20	20

The docking approach was carefully planned to provide detailed insights into protein–ligand interactions. Autodock Vina V 1.2.6 was used for molecular docking (40, 41). To identify the most efficient binding poses and assess reproducibility, parameters were optimized across multiple docking trials with varying grid sizes and tools. Docking parameters included a grid spacing of 0.375 Å num_modes (9), energy range (3), and exhaustiveness (16), along with additional default settings. The reliability and predictive accuracy of the docking protocol were validated by redocking lactate dehydrogenase (LDH, PDB ID: 7DBJ), which was the only target with a native cocrystallized ligand (H1U). Root-mean-square deviation (RMSD) was calculated between the calculated lowest-energy docking pose and the experimental X-ray crystallographic conformation. For the remaining targets, CK-MB (3B6R), cTnT (1J1D), and cTnI (7SC2), redocking validation was not mathematically possible, as these are apo proteins lacking native cocrystal ligands.

Docking outcomes were visualized using Biovia Discovery Studio and Maestro 13.9 Academic Edition 7 for structural examination. The protein ligand interaction profiler (PLIP) (https://plip-tool.biotec.tu-dresden.de/plip-web/plip/index) was utilized to analyze binding interactions (43) (Table 3).

**Table 3:** Comparison of standard values and retrieved protein data for validation of proteins selected for docking study.

Parameters	Target	Protein ID	Method of experiment	Mutation	Resolution	wwPDB validation	Cocrystal ligand	Ramchandran plot (by PROCHECK server) residues in favored + allowed regions (%)
Details	Standards	-	X-Ray diffraction	No	Near about 3.00 Å	Better	-	>88
	CK-MB	3B6R	X-Ray diffraction	No	2	Better	Absent	99.80
	LDH	7DBJ	X-Ray diffraction	No	1.55	Better	H1U	99.50
	cTnT	1J1D	X-Ray diffraction	No	2.61	Better	Absent	100.00
	cTnI	7SC2	X-Ray diffraction	No	1.81	Better	Absent	100.00

### Molecular dynamics simulation

MD simulations were performed using Schrödinger’s Desmond 2020.1, LLC on the docked complexes of 3B6R and 7DBJ with aegeline, designated as 3B6R_Aegeline and 7DBJ_Aegeline, respectively. The OPLS-2005 force field (44–46) was used along with an explicit solvent modeled by TIP3P molecules of water inside a (47) periodic boundary box of dimensions 10 Å×10 Å×10 Å. To neutralize the system, 0.15 M Na + ions were added, along with NaCl, to replicate physiological conditions. To maintain the protein–ligand complexes, the system was initially equilibrated for 10 ns using an NVT (Constant Number of particles, Volume, and Temperature ensemble) ensemble. The Nose–Hoover chain coupling scheme (48) thermostat, with a relaxation time of 1.0 ps and a pressure of 1 bar, was then used for equilibration and minimization under an NPT (Constant Number of particles, Pressure, and Temperature ensemble) ensemble for 12 ns. A time step of two fs was chosen for simulations. The Martyna–Tuckerman–Klein barostat was used for pressure control, with a relaxation time of 2 ps (49). Long-range electrostatic interactions with fixed 9 Å were calculated using the particle mesh Ewald method (50), and a radius was fixed for coulomb interactions. Production MD runs were performed for 100 ns. RMSD, radius of gyration (Rg), root-mean-square fluctuation (RMSF), and the quantity of hydrogen (H-bonds), salt bridges, and solvent accessible surface area (SASA) throughout the simulations were used to evaluate stability. Dynamic cross-correlation matrices, PCA, and free energy landscapes were calculated from the MD trajectories of 1NME-Acemannan and 6CM4-Acemannan. Dynamic cross-correlation matrix (DCCM) was calculated using a Python script, and PCA and free energy landscapes were determined using the Geo_measure tool (51).

### Analysis of binding free energy

The generalized born surface area (MM-GBSA) method in conjunction with molecular mechanics was used to compute the binding free energies of the ligand–protein complexes. The Prime MM-GBSA binding free energy was evaluated using the Python script thermal_mmbsa.py, which analyzed the last 50 frames of the MD simulation trajectory at a sampling frequency of one frame per analysis. The total binding energy (kcal/mol) was computed by summing contributions from individual energies, such as hydrogen bonding, coulombic, covalent, Van der Waals, self-contact, lipophilic, and solvation energies, of both protein and ligand. The formula for calculating *Δ*Gbind was adopted from previous work (52).

### Statistical analysis

Data were analyzed using Windows-based software (GraphPad Prism 8.0.2). Findings were expressed as mean ± SEM. The Shapiro–Wilk test was performed for normally distributed data. Multiple regression analysis and one-way analysis of variance (ANOVA), including Tukey’s *post hoc* test, were used to evaluate differences among the items and examine variance distributions.

## Results

### Impact of aegeline on body weight

Body weight did not differ significantly among the experimental groups at the beginning of the study [*F*(3,20) = 0.3999, *P* = 0.7546] or before myocardial injury induction [*F*(3,20) = 0.4306, *P* = 0.7334], indicating comparable baseline characteristics. As shown in Figure 1, ISO administration led to a significant decrease in average body weight compared to the normal control group. Treatment with aegeline reduced the loss of body weight in ISO-treated rats, showing an improvement over the disease control group, though the difference was not statistically significant. The effect was more pronounced in the higher-dose aegeline group, suggesting a protective role against ISO-induced systemic deterioration.

**Figure 1:**
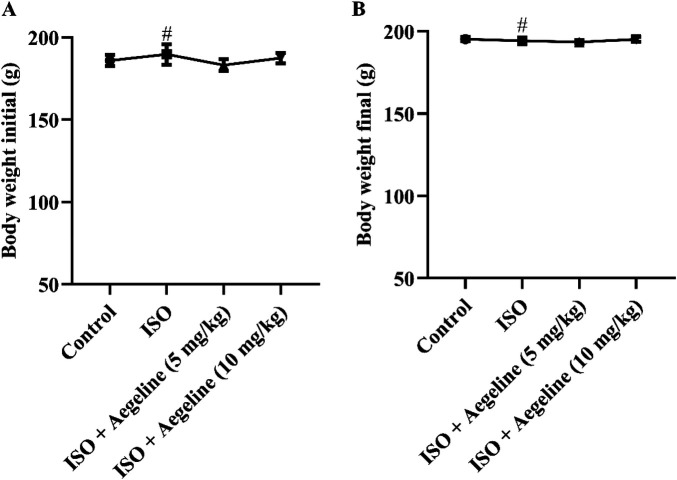
Effects of aegeline on body weight in experimental rats. **(A)** Body weight before treatment and **(B)** body weight after treatment. Data are presented as mean ± SEM (*n* = 6). Statistical significance was determined using one-way ANOVA followed by Tukey’s *post hoc* test. #*P* < 0.05 vs. the control group.

### Impacts of aegeline on cardiac injury biomarkers

The effects of aegeline on serum cardiac injury biomarkers are presented in Figures 2A–D. ISO administration significantly increased serum LDH, CK-MB, cTnT, and cTnI levels compared with the normal control group, indicating substantial myocardial damage. Treatment with aegeline at doses of 5 and 10 mg/kg significantly reduced LDH [*F*(3,20) = 81.33, *P* < 0.0001], CK-MB [*F*(3,20) = 56.57, *P* < 0.0001], cTnT [*F*(3,20) = 11.96, *P* = 0.0001], and cTnI [*F*(3,20) = 13.20, *P* < 0.0001] compared with the ISO control group. As illustrated in Figure 2, aegeline restored these biomarkers to normal values, indicating attenuation of myocardial injury and preservation of cardiac integrity.

**Figure 2:**
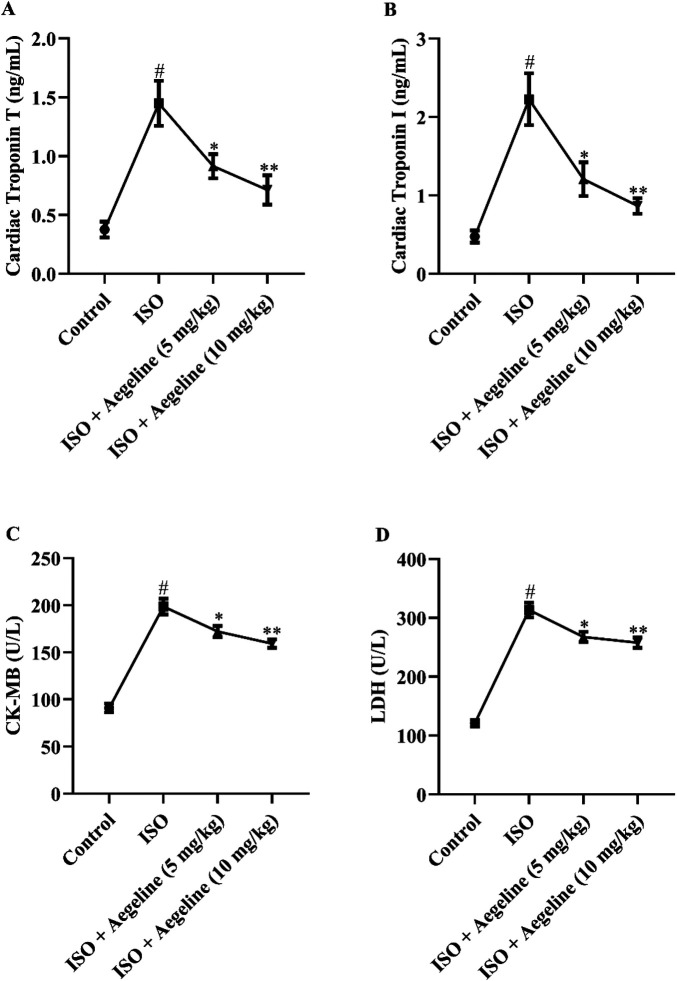
**(A–D)** Effects of aegeline on cardiac injury biomarkers in isoproterenol-induced myocardial infarction. **(A)** Cardiac troponin T (cTnT), **(B)** cardiac troponin I (cTnI), **(C)** creatine kinase-MB (CK-MB), and **(D)** lactate dehydrogenase (LDH). Data are presented as mean ± SEM (*n* = 6). Statistical significance was determined using one-way ANOVA followed by Tukey’s *post hoc* test. #*P* < 0.05 vs. the control group; *P < 0.05 and **P < 0.01 vs. the ISO group.

### Effects of aegeline on aldosterone and cortisol

Figures 3A,B show the effects of aegeline on aldosterone and cortisol levels. ISO-treated rats exhibited significantly elevated concentrations of both hormones compared with normal controls, reflecting neurohormonal activation associated with myocardial injury. Treatment with aegeline significantly reduced aldosterone [*F*(3,20) = 35.84, *P* < 0.0001] and cortisol levels compared with the disease control group. The reduction was more evident in rats receiving the higher dose of aegeline, suggesting improvement in the hormonal disturbances associated with MI.

**Figure 3:**
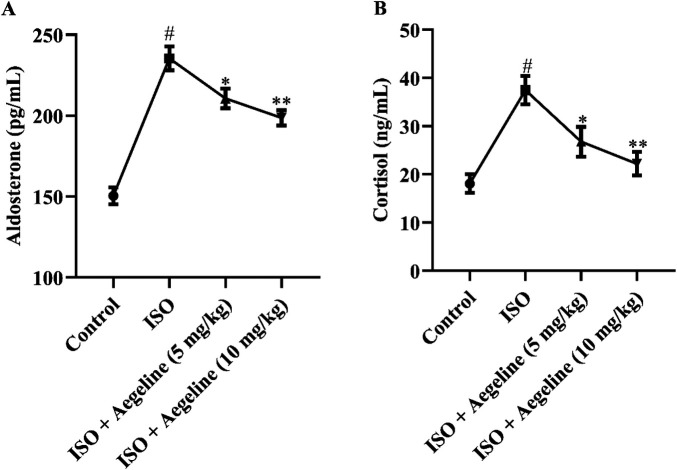
Effects of aegeline on neurohormonal markers in isoproterenol-induced myocardial infarction. **(A)** Aldosterone and **(B)** cortisol levels. Data are presented as mean ± SEM (*n* = 6). Statistical significance was determined using one-way ANOVA followed by Tukey’s *post hoc* test. #*P* < 0.05 vs. the control group; *P < 0.05 and **P < 0.01 vs. the ISO group.

### Impact of aegeline on antioxidant status, oxidative stress, and nitrosative stress markers

The effects of aegeline on oxidative and nitrosative stress parameters are shown in Figures 4A–E. ISO administration significantly impaired antioxidant defenses, as evidenced by reduced GSH and CAT levels, while markedly increasing MDA, ROS, and NOx levels compared with the normal control group. Aegeline treatment significantly increased GSH [*F*(3,20) = 5.324, *P* = 0.0073] and CAT [*F*(3,20) = 22.45, *P* < 0.0001] levels while reducing ROS [*F*(3,20) = 10.459, *P* = 0.0022], NOx [*F*(3,20) = 9.603, *P* = 0.0004], and MDA [*F*(3,20) = 7.894, *P* = 0.0011] levels compared with the ISO group. As illustrated in Figure 4, these findings demonstrate that aegeline effectively mitigated oxidative and nitrosative stress induced by ISO.

**Figure 4:**
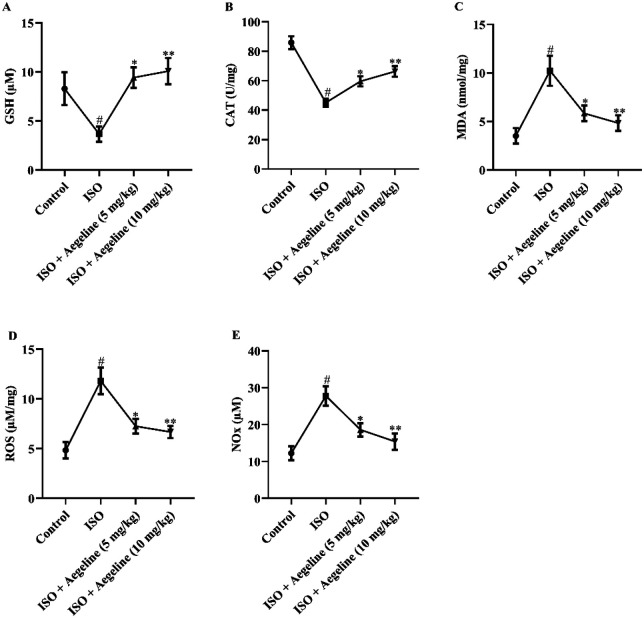
Effects of aegeline on antioxidant status, oxidative stress, and nitrosative stress markers in isoproterenol-induced myocardial infarction. **(A)** Reduced glutathione (GSH), **(B)** catalase (CAT), **(C)** malondialdehyde (MDA), **(D)** reactive oxygen species (ROS), and **(E)** total nitrite/nitrate (NOx). Data are presented as mean ± SEM (*n* = 6). Statistical significance was determined using one-way ANOVA followed by Tukey’s *post hoc* test. #*P* < 0.05 vs. the control group; *P < 0.05 and **P < 0.01 vs. the ISO group.

### Impact of aegeline on inflammatory markers

The effects of aegeline on inflammatory mediators are presented in Figures 5A–D. ISO administration caused a significant increase in TNF-α, IL-1β, IL-6, and NF-*κ*B levels compared with normal control rats, confirming the development of a pronounced inflammatory response. Treatment with aegeline significantly reduced TNF-α [*F*(3,20) = 41.18, *P* < 0.0001], IL-1β [F(3,20) = 42.43, *P* < 0.0001], IL-6 [F(3,20) = 32.75, *P* < 0.0001], and NF-*κ*B [F(3,20) = 7.129, *P* = 0.0019] levels compared with the ISO group. As shown in Figure 5, the findings demonstrate suppression of inflammatory mediators, indicating that aegeline exerts cardioprotective effects, at least in part, by modulating inflammatory pathways.

**Figure 5:**
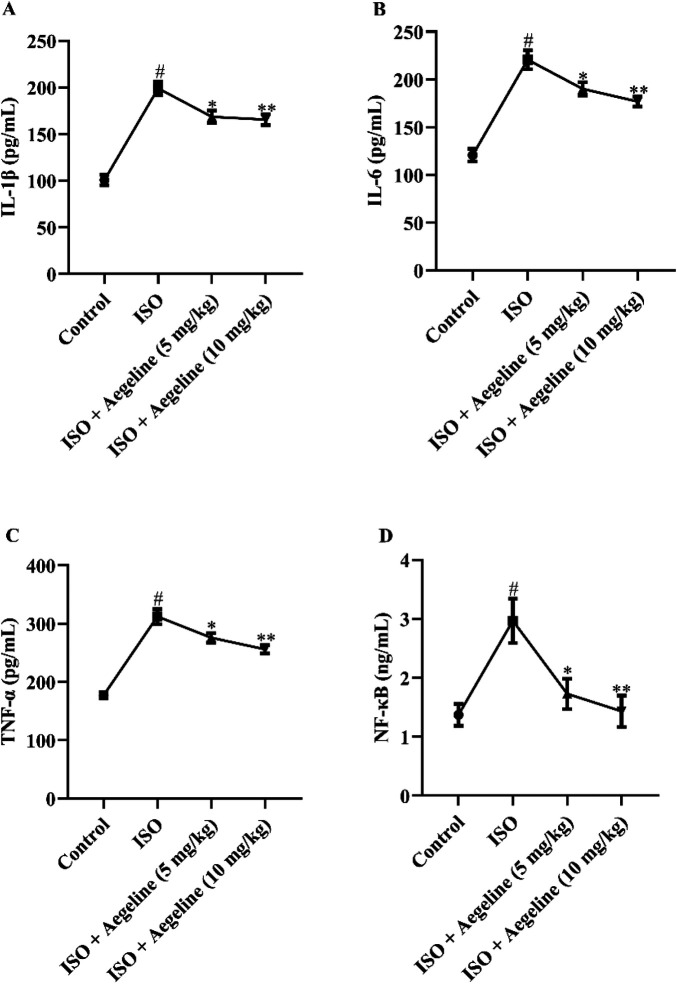
Effects of aegeline on inflammatory mediators in isoproterenol-induced myocardial infarction. **(A)** Interleukin-1β (IL-1β), **(B)** interleukin-6 (IL-6), **(C)** tumor necrosis factor-α (TNF-α), and **(D)** nuclear factor kappa B (NF-*κ*B). Data are presented as mean ± SEM (*n* = 6). Statistical significance was determined using one-way ANOVA followed by Tukey’s *post hoc* test. #*P* < 0.05 vs. the control group; *P < 0.05 and **P < 0.01 vs. the ISO group.

### Impact of aegeline on hepatic and renal function

The effects of aegeline on hepatic and renal function markers are shown in Figures 6A–D. ISO administration significantly elevated serum ALT, AST, urea, and creatinine levels compared with the normal control group, suggesting hepatic and renal dysfunction secondary to myocardial injury. Treatment with aegeline significantly reduced ALT [*F*(3,20) = 24.35, *P* < 0.0001], AST [*F*(3,20) = 24.65, *P* < 0.0001], creatinine [*F*(3,20) = 8.505, *P* = 0.0008], and urea [*F*(3,20) = 9.502, *P* = 0.0004] levels compared with ISO-treated rats. These findings indicate that aegeline ameliorated the systemic biochemical disturbances associated with ISO-induced MI.

**Figure 6:**
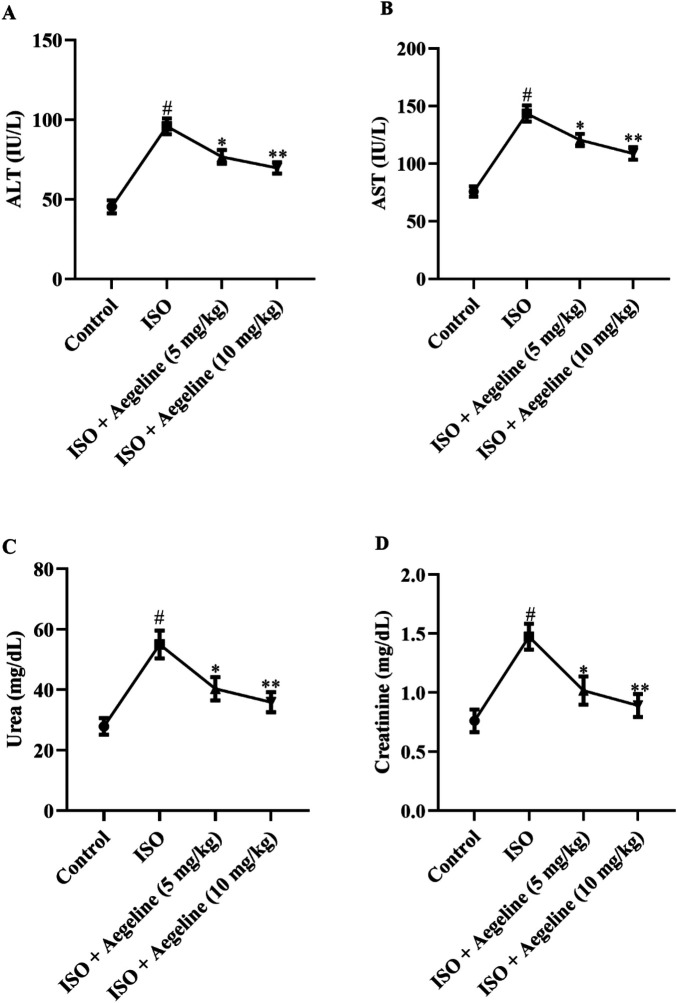
Effects of aegeline on hepatic and renal function markers in isoproterenol-induced myocardial infarction. **(A)** Aspartate aminotransferase (AST), **(B)** alanine aminotransferase (ALT), **(C)** urea, and **(D)** creatinine. Data are presented as mean ± SEM (*n* = 6). Statistical significance was determined using one-way ANOVA followed by Tukey’s *post hoc* test. #*P* < 0.05 vs. the control group; *P < 0.05 and **P < 0.01 vs. the ISO group.

### Impact of aegeline on apoptosis markers

The effect of aegeline on apoptosis is shown in Figure 7. Caspase-3 levels were significantly elevated in the ISO-treated group compared with the normal control group, indicating enhanced apoptotic activity in myocardial tissue. Administration of aegeline significantly reduced caspase-3 expression [*F*(3,20) = 10.75, *P* = 0.0002] compared with the disease control group. As shown in Figure 7, the findings demonstrate that aegeline suppressed apoptosis associated with myocardial injury, thereby contributing to its cardioprotective effect.

**Figure 7:**
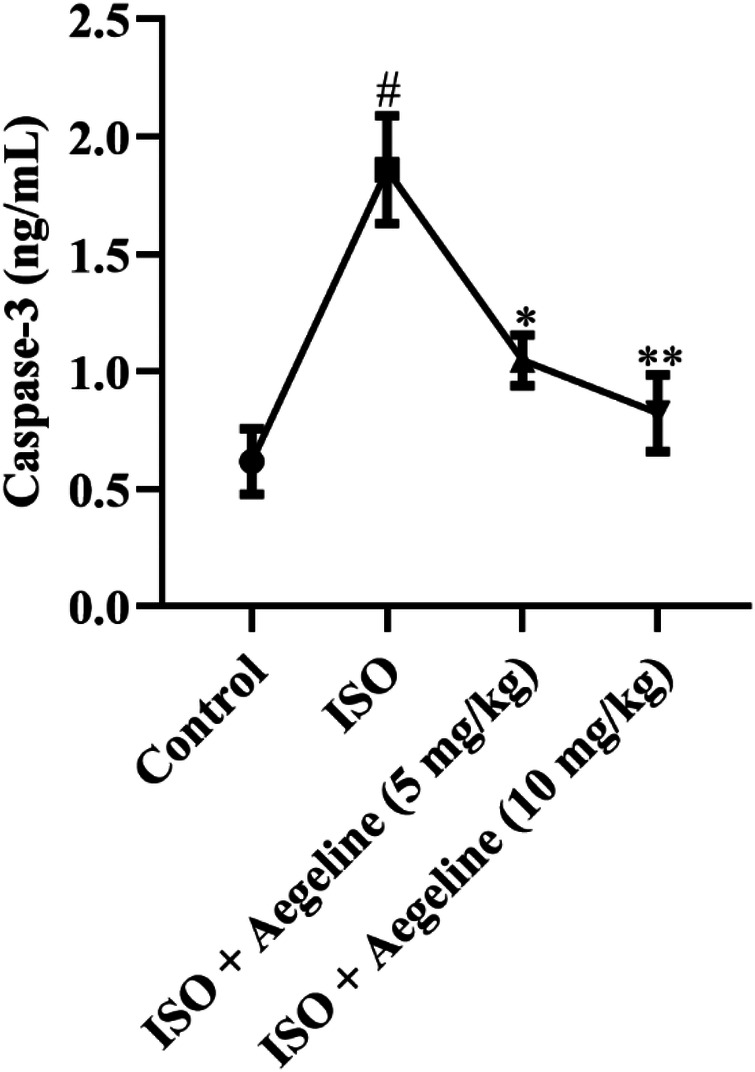
Effects of aegeline on caspase-3 levels in isoproterenol-induced myocardial infarction. Caspase-3 levels were significantly increased in the ISO group compared with the control group, indicating enhanced apoptotic activity following myocardial injury. Treatment with aegeline at doses of 5 and 10 mg/kg significantly reduced caspase-3 levels compared with the ISO group. Data are presented as mean ± SEM (*n* = 6). Statistical analysis was performed using one-way ANOVA followed by Tukey’s *post hoc* test. #*P* < 0.05 vs. control; *P < 0.05 and **P < 0.01 vs. ISO.

### Impact of aegeline on heart histopathology

Representative histopathological images and quantitative injury scores are presented in Figures 8A–E. The myocardium of the normal control group (Figure 8A) exhibited well-organized cardiac muscle fibers with preserved cytoplasmic striations and uniformly distributed nuclei. In contrast, the ISO-treated group (Figure 8B) showed extensive myocardial damage characterized by myocardial fiber necrosis, interstitial edema, vascular congestion, and marked mononuclear inflammatory cell infiltration. Rats treated with aegeline at 5 mg/kg (Figure 8C) displayed moderate improvement in myocardial architecture, with reduced inflammatory cell infiltration and less disruption of myocardial fibers. Treatment with aegeline at 10 mg/kg (Figure 8D) produced greater histological protection, as evidenced by near-normal myocardial architecture, minimal inflammatory infiltration, and preservation of myocardial fibers.

**Figure 8:**
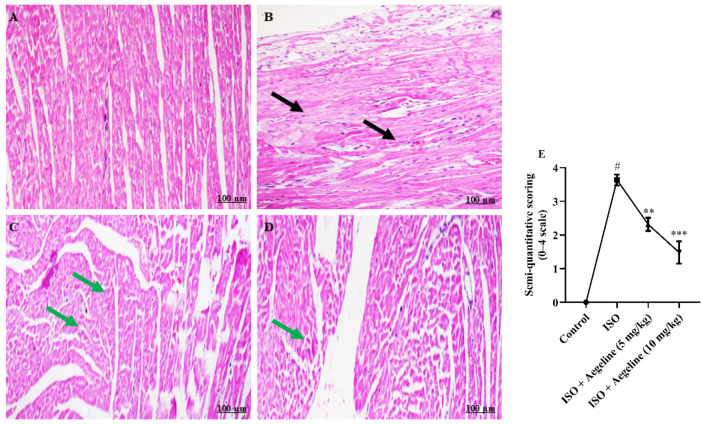
Effects of aegeline on myocardial histopathological alterations in isoproterenol-induced myocardial infarction. Representative hematoxylin and eosin (H&E)-stained myocardial sections (200× magnification; scale ba*r* = 100 μm). **(A)** Control group showing normal myocardial architecture with well-organized cardiac muscle fibers, intact cytoplasmic striations, and uniformly distributed nuclei. **(B)** ISO-induced myocardial infarction group showing marked myocardial injury characterized by endocardial edema, myofibrillar degeneration, and extensive mononuclear inflammatory cell infiltration (black arrows). **(C)** ISO + aegeline (5 mg/kg) group showing partial preservation of myocardial architecture with moderate inflammatory cell infiltration and reduced tissue damage (green arrows). **(D)** ISO + aegeline (10 mg/kg) group showing near-normal myocardial morphology with minimal inflammatory cell infiltration and preserved myocardial fibers (green arrow). **(E)** Quantitative histopathological injury scores were determined using a blinded semiquantitative scoring system (0–4 scale). Data are presented as mean ± SEM (*n* = 6). Statistical analysis was performed using one-way ANOVA followed by Tukey’s *post hoc* test. #*P* < 0.05 vs. control group; **P < 0.01 and ***P < 0.001 vs. ISO-treated group.

Quantitative histopathological assessment (Figure 8E) further confirmed these observations. The ISO-treated group exhibited a significantly higher myocardial injury score than the normal control group (*P* < 0.005). Both aegeline-treated groups showed significantly lower injury scores than the ISO group, with preservation of myocardial structure and reduced inflammatory changes. One-way ANOVA demonstrated a significant overall treatment effect on histopathological scores [*F*(3,20) = 53.19, *P* < 0.0001], supporting aegeline’s cardioprotective effect against ISO-induced myocardial injury.

### Molecular docking

Autodock Vina V. 1.2.6 was used to perform a docking study on CK-MB (3B6R), LDH (7DBJ), cTnT (1J1D), and cTnI (7SC2). The types of interactions that take place at the particular target’s active site and the binding energy affinities were examined in the docking result (Supplementary Figure 1).

The detailed molecular interactions of aegeline with CK-MB (3B6R), LDH (7DBJ), cTnT (1J1D), and cTnI (7SC2) were retrieved using PLIP (Table 4). Based on docking scores, aegeline exhibited the best binding affinity for CK-MB (3B6R) through hydrophobic, hydrogen-bonding, and salt bridge interactions (Figure 9).

**Table 4:** List of interactions between ligand and proteins identified using PLIP.

Sr. no.	Name of complex	Binding energy (kcal/mol)	Type of interaction: residue ID (distance Å)
1	3B6R-AEGELINE	−8.306	Hydrophobic interactions: ARG130B (3.57), HIS191B (3.7), TRP228B (3.59)
			Hydrogen bonds: HIS191B (2.29), ASP195B (2.9)
			π-Stacking: HIS296B (3.81)
2	7DBJ-AEGELINE	−6.525	Hydrophobic interactions: ASN206A (3.84), ASN206C (3.78), LYS308A (3.67), LYS310A (3.6)
			Hydrogen bonds: SER203A (3.26), SER203A (2.57)
			π-Cation Interactions: LYS308A (5.9)
3	1J1D-AEGELINE	−5.853	Hydrophobic interactions: TYR150D (3.59), GLU260E (3.43), VAL263E (3.78)
			Hydrogen bonds: ARG267E (2.02), ARG267E (3.07), ARG267E (2.62)
4	7SC2-AEGELINE	−6.364	Hydrophobic interactions: ALA23A (3.89), PHE27A (3.51), PHE27A (3.77), PHE27A (3.64), ILE36A (3.6), LEU41A (3.64), VAL44A (3.56), LEU48A (3.93), MET60A (3.95), ILE61A (3.39), VAL72A (3.64)

**Figure 9:**
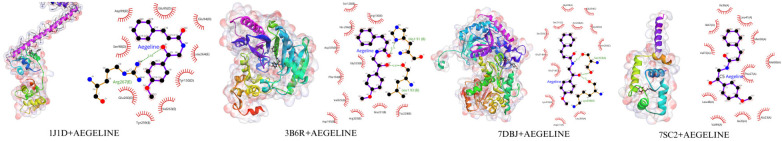
Two-dimensional and three-dimensional molecular interactions of the ligand aegeline with 3B6R, 7DBJ, 1J1D, and 7SC2.

### Molecular dynamics simulation (MDS)

The stability and convergence of 3B6R_Aegeline, 7DBJ_Aegeline complexes were assessed using MDS. Supplementary Figure 2A shows an analysis of the 3B6R_aegeline complex’s RMSD over a 100-ns simulation period. During the initial 10 ns, the RMSD values rose sharply to ∼2.5 Å, indicating early conformational changes in the complex. Beyond 20 ns, the RMSD fluctuated with an average value of 3.10 Å and gradually stabilized above 3.5 Å. The relatively small fluctuations and stabilization indicate that the 3B6R_Aegeline complex retained stable conformation. Although the complex exhibited an RMSD above 3.5 Å after equilibration, the trajectory reached a stable plateau after approximately 20 ns, indicating that the protein-ligand complex achieved a stable dynamic equilibrium in solution rather than undergoing continuous structural destabilization. Supplementary Figure 2B presents RMSD analysis of the 7DBJ_aegeline complex during a 100-ns simulation period. The RMSD reached 4-5 Å during first 15 ns, followed by pronounced fluctuations. Notable peaks were observed around 20 and 50–60 ns with RMSD above 6.5 Å, indicating conformational flexibility within binding pocket. However, the RMSD stabilized with an average value of 4.98 Å, higher than the 3B6R_Aegeline complex, indicating less structural stability. The high RMSD indicates extensive domain plasticity and flexible loop motions, rather than complicated dissociation or unfolding. Supplementary Figure 2C presents the RMSF analysis of 3B6R_aegeline. The RMSF value exhibited higher peaks at the terminal end, indicating terminal flexibility. Within the structured core, residues exhibited minimal fluctuations, below 3 Å, suggesting stable interactions. Higher fluctuations were observed in residue numbers 0–9, 323, and 371–375, indicating flexibility in these regions. Supplementary Figure 2D presents the RMSF profile of the 7DBJ_aegeline complex, which exhibited pronounced fluctuations, indicating higher flexibility at residue numbers 0–16, 53–55, 67, 69–70, 333–349, and 578, with RMSF above 3 Å. Beyond residue 30, fluctuations were reduced, with average RMSF 1.71 Å, characterizing a stable region. Supplementary Figure 2E shows the 3B6R_aegeline radius of gyration (Rg) analysis, which was carried out to assess the compactness and structural stability of the protein. The Rg value of 3B6R_Aegeline fluctuated between 21 and 22.09 Å, with minor variations. This relatively stable Rg indicates that the protein maintained consistent compactness and structural stability. Supplementary Figure 2F shows the 7DBJ_aegeline’s Rg analysis, which fluctuated between 4.9 Å and 5.62 Å, with frequent but small variations. However, minor dips were observed between 30 and 40 ns, around 5 Å within a narrow range, reflecting overall structural compactness. Supplementary Figure 3A shows how many hydrogen bonds (H-bonds) were formed in 3B6R_aegeline and 1NME_Aegeline. Between 0 and 4, the average number of hydrogen bonds was 1.67, with an average of 1.67 being maintained during simulation. This suggests that hydrogen bonding contributed significantly to stabilization of the complex. Supplementary Figure 3B shows that a number of hydrogen bonds were formed in 7DBJ_aegeline, ranging between 0 and 4, with an initial higher fluctuation followed by stabilization around 1–2 hydrogen bonds. This indicates that the ligand initially formed strong hydrogen bond interactions with protein, which decreased slightly over time. Supplementary Figure 3C-D illustrates the SASA analysis, emphasizing the stability and conformational changes of aegeline upon binding to proteins 3B6R and 7DBJ. The reduction in SASA upon aegeline binding to both 3B6R and 7DBJ protein indicates a substantial conformational change, leading to decreased solvent exposure of the protein surface. In an unbound state, both proteins displayed higher SASA values and large fluctuations, suggesting flexible and solvent-exposed conformations. The bound state, however, restricted conformational flexibility, resulting in lower SASA values.

Protein–ligand interactions during the 100-ns MD simulations were monitored and summarized by using stacked colored bar graphs, normalized over the entire simulation trajectory. These interactions were classified into water bridges, ionic interactions, hydrophobic interactions, and H bonds. When a protein–ligand complex exhibited multiple contacts of specific interaction types, the corresponding values remained ≥ 1. Supplementary Figures 4A,B show bar graphs of the fractions of these interaction types mapped to the residues involved over the 100-ns simulation period. It reveals that hydrogen bonding, ionic interactions, and water bridges yield high interaction fractions for the protein–ligand complexes 3B6R_Aegeline and 7DBJ_Aegeline.

Supplementary Figure 4C shows the 3B6R_Aegeline complex’s percentage of protein–ligand interactions. Aegeline formed a strong hydrogen bond with ASP335 (138%). Water-mediated interactions were observed with ARG132 (96%). HIS191 (14%) interacted via hydrophobic interaction, and ARG292 (65%) interacted via ionic interaction. Supplementary Figure 4D shows the percentage of protein–ligand interactions for the 7DBJ_Aegeline complex. GLU214 (13%), GLY209 (22%), SER211 (12%), and LYS310 (18%) interacted via hydrogen bonding. LYS310 (26%) and LYS308 (10%) interacted via ionic interactions. GLU214 (28%) interacted via water bridges.

### Principal component analysis

The principal component analysis (PCA) of MD trajectories for the two complexes, 3B6R–aegeline and 7DBJ–aegeline, provided critical insights into their conformational landscapes, stability, and dynamic behavior during the simulation period. In the case of 3B6R–aegeline**,** the PCA plots (top row) reveal that the PC1 and PC2, the first two principal components, were responsible for a significant proportion of the overall motion, with PC1 alone capturing nearly 89.91% of the variance, while PC2 contributed an additional 5.45%. The significant dominance of PC1 suggests that the conformational space of 3B6R–aegeline is largely driven by a single collective motion, reflecting a relatively restricted but stable dynamic pathway. The clustering pattern shows a progressive shift of conformations over the trajectory, with frames colored from red to blue, indicating a directional and somewhat constrained conformational transition rather than random fluctuations. Interestingly, when higher-order components (PC9 vs. PC10) are plotted, the distribution becomes more scattered and isotropic, contributing negligibly (<0.1%) to the variance, confirming that major conformational changes are adequately captured by the first few PCs. The 7DBJ–aegeline complex (bottom row) displays a more diverse conformational landscape. Here, PC1 accounts for 72.01% of the variance, while PC2 and PC3 contribute 18.88% and 4.23%, respectively, reflecting a more distributed motion contribution compared to 3B6R. The trajectory points are more widely scattered across the PCA space, forming multiple clusters, suggesting that 7DBJ–aegeline explores a broader conformational space with more transitions among metastable states. This indicates higher flexibility or plasticity in the 7DBJ binding environment. Furthermore, the color-coded progression from red to blue frames shows a more cyclical, overlapping spread, suggesting repeated exploration of conformations rather than a one-way shift, as seen in 3B6R. When comparing both complexes, the data indicated that 3B6R–aegeline adopted a more stable, directed conformational adaptation dominated by a single principal motion, while 7DBJ–aegeline explored a wider conformational space, with multiple significant motions contributing, reflecting greater flexibility and potential adaptive binding dynamics. From a functional standpoint, this could imply that aegeline binding in 3B6R stabilizes the protein in a specific state with limited fluctuations, potentially favoring stable inhibitory interactions. In contrast, the more dynamic 7DBJ–aegeline complex suggests less conformational restriction, possibly allowing plasticity in binding interactions but at the cost of reduced stability. Overall, the PCA analysis underscores that 3B6R–aegeline is the more stable complex**,** while 7DBJ–aegeline demonstrates enhanced conformational diversity and flexibility; these differences may have important implications for their comparative biological activities (Supplementary Figure 5).

### Dynamic cross-correlation

The DCCM analysis of MD trajectories for 3B6R–aegeline and 7DBJ–aegeline complexes provided a detailed overview of the cooperative and antagonistic motions among protein residues during ligand binding. In the 3B6R–aegeline complex (left panel), the correlation map indicated a balanced distribution of favorably connected motions (blue regions) and anticorrelated motions (red regions), suggesting well-organized global dynamics. Residues between 50–150 and 200–300 exhibited strong positive correlations, reflecting collective domain movements that may stabilize aegeline binding. Meanwhile, localized anticorrelations, particularly between residues 100–200 and 250–350, suggested flexibility in loop and linker regions that contribute to subtle local adjustments around the binding pocket. The predominance of blue patches along the diagonal implied that large segments of residues were moving in synchrony. This indicates that 3B6R underwent stable global motions with localized fluctuations, a pattern consistent with a ligand-induced rigidification of the protein core. In contrast, the 7DBJ–aegeline complex (right panel) revealed a more heterogeneous correlation landscape, with large contiguous blocks of positive and negative correlations. Strong positive correlations were evident between residues 150–300 and 350–500, suggesting large-scale domain motions contributing to global flexibility. At the same time, extensive anticorrelated motions appeared between N-terminal residues (50–200) and C-terminal segments (400–600), indicating that different structural domains move in opposing directions, thereby increasing dynamic plasticity. The diagonal streaks of both strong blue and red regions suggested that local motions within secondary structure elements were not uniformly cooperative, but rather influenced by ligand binding that induces local flexibility and long-range communication. The wider distribution of correlation and anticorrelation in 7DBJ-aegeline indicates that aegeline binding does not stabilise the protein as rigidly as in 3B6R, but instead encourages overall conformational flexibility. Taken together, the DCCM comparison highlights that 3B6R–aegeline demonstrated more synchronized residue motions with compact global stability, favoring a stable binding conformation, while 7DBJ–aegeline exhibited more distributed and flexible residue correlations, indicative of complex global and local motions that may enable conformational sampling of multiple binding states. Functionally, this suggests that the 3B6R complex may provide a more stable inhibitory platform for aegeline. In contrast, the 7DBJ complex retains structural flexibility that could facilitate adaptive binding interactions but potentially at the cost of reduced overall stability (Supplementary Figure 6).

### Free energy landscape

The free energy landscape (FEL) plots illustrate the conformational stability and energy minima of two protein–ligand complexes across the reaction coordinates of RMSD (*x*-axis) and radius of gyration (*y*-axis), with Gibbs free energy shown as a color gradient. In both complexes, the low-energy conformations are depicted by the deep blue basins, representing the most stable states achieved during the molecular dynamics simulations. For the first complex (top panels), the FEL reveals a single well-defined basin around an RMSD range of ∼3.5–4.0 Å and a radius of gyration of ∼21.7–21.9 Å, suggesting a compact and energetically favorable conformation. The narrowness and depth of this basin indicate that the complex attains a relatively rigid, stable structure with limited conformational fluctuations, reinforcing its strong binding affinity and structural integrity. In contrast, the second complex (bottom panels) displays a broader and more scattered energy distribution with multiple basins, primarily centered at RMSD values of ∼4.5–6.0 Å and a radius of gyration of ∼28.6–29.2 Å. Although the presence of blue regions indicates stable conformations, the spread across wider RMSD and Rg values suggests greater structural flexibility and higher conformational sampling, which often correlates with reduced rigidity and potentially less stable binding than the first complex. Importantly, the blue basin in the first complex is sharper and deeper, signifying a dominant global minimum that the system favors throughout the trajectory, while the second complex shows several shallow basins that may correspond to local minima, implying dynamic interconversions between metastable states. Thus, when comparing the two, the first complex demonstrates superior structural stability and stronger thermodynamic favorability, as evidenced by its concentrated and energetically deeper free energy basin, while the second complex, though stable in certain conformations, exhibits higher degrees of flexibility and weaker stabilization. Overall, the FEL comparison underscores that the first complex is more stable, with its blue basin signifying a well-defined native-like state, whereas the second complex is relatively less stable due to its distributed minima and higher conformational heterogeneity (Supplementary Figure 7).

## Discussion

In recent decades, the growing number of patients with CVD, particularly MI, has challenged the global healthcare structure due to its high mortality burden (53). The present study expands the current understanding of aegeline by demonstrating its protective effects against ISO-induced MI in diabetic rats. While earlier investigations primarily examined the broad pharmacological activities of *Aegle marmelos* and its constituents, this study provides a more comprehensive evaluation of aegeline by assessing cardiac injury biomarkers, oxidative stress, inflammation, apoptosis, histopathological alterations, and computational analyses. Collectively, these findings provide insights into the mechanisms that may contribute to aegeline’s cardioprotective actions.

It should be noted that CK-MB, LDH, cTnT, and cTnI are well-established indicators of myocardial injury and are not considered therapeutic targets. Accordingly, the molecular docking and molecular dynamics studies were performed to explore the potential interactions of aegeline with proteins associated with cardiac injury, rather than to demonstrate direct target modulation. Therefore, the computational results should be seen as supportive and exploratory. The evidence for cardioprotection mainly arises from improvements in cardiac biomarkers, oxidative stress status, inflammatory mediators, apoptotic markers, and myocardial histopathology observed in the experimental model. However, the degree to which these molecular and structural improvements translate into better cardiac function still needs to be determined. Future evaluations of hemodynamic and echocardiographic parameters would offer a more complete assessment of aegeline’s cardioprotective effects and help determine its potential role in managing myocardial injury.

ISO, a synthetic catecholamine and *β*-adrenergic agonist, regulates myocardial metabolism and contractility. It is a crucial component of a standard model for studying the potential benefits of various medications on heart function (54). ISO causes cardiac necrosis through multiple processes, including greater oxygen consumption, calcium overload and accretion, reformed cardiac metabolism of cells, elevated myocardium cAMP levels, increased lipid peroxides, intracellular acidosis, changed membrane permeability, and a disrupted electrolyte milieu (55). The myocardial necrotic rat model’s heart exhibits pathophysiological and morphological abnormalities that are similar to those observed in human MI. ISO-induced MI causes alterations in integrity and/or permeability of the plasma membrane in response to *β*-adrenergic stimulus, resulting in the release of cardiac enzymes. The *β*-agonist may have caused injury to the sarcolemma, resulting in leakage. In this study, we established that ISO administration in rats was related to myocardial damage as recognized by a substantial increase of cardiac damage indicators in the ISO control group. In addition, it triggered alterations in lipid peroxidation, endogenous antioxidants and nitrate stress markers, inflammatory markers, and inhibition of apoptotic expression.

Cardiac troponins are low-molecular-weight proteins that form part of the contractile mechanism of muscle myofibrils. They are highly sensitive and specific markers of cardiomyocyte injury in healthy individuals, are not normally present in the blood, and are only released during myocardial necrosis (56). In this research, augmented levels of cardiac troponin were attributed to the ISO-induced myocardial impairment, which was reversed by aegeline. Aegeline is thought to preserve the contractile apparatus’s structural and functional integrity, averting cardiac damage. The myocardium shows diagnostic signs of MI and releases them into the extracellular fluids after injury (57). The specific markers of tissue injury are myocardial enzymes and macromolecules, which are found in injured tissues due to their tissue-specific profiles and biochemical catalytic capabilities. The bloodstream obtains cytosolic enzymes, including LDH, CK-MB, AST, and ALT, through cell membrane permeability or rupture. The levels of cellular enzymes in the bloodstream signal changes in plasma membrane structure and accessibility.

In the current investigation, rats injected with ISO revealed substantial increase in all these cardiac markers, in keeping with the findings of earlier data, demonstrating ISO-induced MI and plasma membrane permeability (58). However, aegeline decreased membrane integrity activity, limiting the leakage of these enzymes. Previous cardioprotective studies using *A. marmelos* fruit extract to reduce oxidative stress and pathological changes following ISO-induced MI corroborated our findings (59). Early measurements of cortisol and aldosterone following acute MI have been linked to medium-term left ventricular remodeling (60). A previous study demonstrated that elevated serum cortisol levels were an impartial interpreter of cardiac measurements in individuals suffering from chronic heart failure (61). In our study, aegeline resulted in a more substantial decline in aldosterone and cortisol levels as directed by the myocardial-salvaging properties of aegeline.

ISO quinone metabolites may also promote oxidative stress, combining with oxygen to produce ROS such as superoxide anions that inhibit the function of antioxidant enzymes (62). Antioxidant enzymes are critical in preventing tissue damage caused by oxygen-free radicals. In the present investigation, aegeline treatment significantly improved lipid peroxidation, antioxidant defense systems, and nitrate stress indicators, as evidenced by increased GSH and CAT, and decreased MDA, ROS, and NOx. Aegeline therapy significantly reversed these changes. Chemical complexes known as antioxidants have the ability to safely interact with free radicals and stop the chain reaction before it damages essential elements (4). Another investigation validated our conclusion that aegeline’s antioxidant activities lessen the production of ROS and increase hydrogen sulfide to prevent high glucose-induced renal cell fibrosis (16).

TNF-α, IL-6, and IL-1β cytokines are pro-inflammatory pointers that play active roles in numerous inflammatory pathways such as NF-*κ*B, JNK, and NOD-like receptor family pyrin domain-containing protein (NLRP) (63). In the pathogenesis of MI, NF-B regulates transcription of several genes, including IL-6 and TNF-α (64, 65). These inflammatory cytokines signaling pathways may encourage the progression of MI. It was observed that ISO-induced animals showed significant changes in IL-1β, IL-6, TNF-α, and NF-kB levels. Our findings demonstrated that, compared to ISO control rats, aegeline reduced IL-1β, IL-6, TNF-α, and NF-kB levels, indicating that it has anti-inflammatory effects that safeguard heart tissue. Our data confirm earlier findings that aegeline mitigated 2,4,6-trinitrobenzene sulfonic acid (TNBS)-induced colitis and reduced colon damage by suppressing transcriptional expression and inflammatory enzymes, most likely via the NFkB-mediated NLRP3 inflammasome pathway (23).

Apoptosis is a carefully delimited cell death process that plays a crucial role in a variety of cardiovascular illnesses, including MI, heart failure, and reperfusion injury. Caspases, crucial enzymes in the initiation and execution of apoptosis, have been recognized as the most promising target for antiapoptotic healing. Caspases are cysteine proteases that play a vital part in the apoptosis lane (66). Apoptosis may be exacerbated by aberrant expression of Bcl-2 and Bax. In the present investigation, ISO increased the mRNA expression of apoptotic indicators such as caspase-3. Other caspases in the apoptotic cascade became phosphorylated as a result of elevated caspase-3 expression. Aegeline treatment reduced caspase-3 expression, indicating that it exerts antiapoptotic effects and provides cardioprotection.

The present study also evaluated the safety of aegeline on vital organs, including the liver and kidneys. Aegeline significantly reduced hepatic and renal markers in the treatment group rats as compared with diseased animals. Our findings demonstrate the potential role of *A. marmelos* leaf extract against cadmium-induced changes in hepatic and renal damage.

The histopathology of healthy rat heart slices demonstrated normal architecture of heart muscle, with no inflammatory infiltration and necrosis. In contrast, ISO-treated rats’ cardiac tissues showed extensive muscle fiber necrosis. Myocardial structural abnormality results in vascular congestion and leukocyte infiltration. As previously stated, substantial histological changes demonstrate the involvement of inflammatory processes and oxidative stress in MI pathogenesis. Compared to ISO-administered rat hearts, the aegeline-treated rat hearts showed no indications of focal necrosis, and the normal morphology of cardiac muscle was well preserved. The improvements in supplementary biochemical characteristics may be regarded as ample indication of the myocardial salvaging effect of aegeline in MI prevention. These histopathological findings are consistent with earlier experimental investigations on MI (59). Another study reported that aegeline treatment in high-cholesterol diet-confronted rats exhibited no signs of anomaly in the tunica intima and medial layers of aortic architecture (17).

Molecular docking revealed that aegeline preferentially binds CK-MB having a binding energy of −8.306 to 8.306 kcal/mol, followed by LDH, cTnI, and cTnT, highlighting its affinity and stability with key proteins involved in myocardial injury. These data align with recent studies employing similar docking tools, where stronger negative binding energies indicate greater inhibitory potential of natural products against disease-related protein targets (52).

MM-GBSA-based binding free energy analyses reported a more favorable *Δ*Gbind for CK-MB-aegeline (−39.92 to 39.92 kcal/mol) compared with LDH–aegeline (−28.73 to 28.73 kcal/mol), consistent with findings that van der Waals and lipophilic parameters predominantly contributed to stabilization, a trend substantiated in recent in silico pharmacological screens (Table 5). PCA further indicated that CK-MB–aegeline’s conformational motions were primarily directed by a single principal axis, indicative of restricted dynamics and stable adaptation, whereas the LDH complex explored broader conformational subspaces (52, 67).

**Table 5:** Binding free energy components for the 3B6R_aegeline and 7DBJ_aegeline complexes calculated by MM-GBSA.

Energies (kcal/mol)	3B6R_Aegeline	7DBJ_Aegeline
*Δ*G_bind_	−39.92 ± 2.40	−28.73 ± 2.92
*Δ*G_bind_Coulomb	−10.91 ± 3.36	−11.31 ± 3.42
*Δ*G_bind_Covalent	3.07 ± 1.24	1.22 ± 0.84
*Δ*G_bind_H_bond_	−1.70 ± 0.13	−0.47 ± 0.26
*Δ*G_bind_Lipo	−13.36 ± 0.65	−8.67 ± 1.18
*Δ*G_bind_Packing	−2.76 ± 0.55	−1.01 ± 0.65
*Δ*G_bind_SolvGB	23.32 ± 1.87	22.13 ± 2.43
*Δ*G_bind_VdW	−37.57 ± 1.76	−30.63 ± 2.19

Dynamic cross-correlation mapping revealed that CK-MB–aegeline featured synchronized residue motions and global stabilization. LDH–aegeline exhibited distributed, flexible interactions, in line with contemporary understanding that ligand-induced protein rigidification underpins drug efficacy. Free energy landscape analysis visualized a deep, confined stability basin for CK-MB–aegeline, supporting the notion of strong conformational stability, with LDH–aegeline presenting broader minima that reflect conformational fluidity (67). Recent studies continue to validate these in silico approaches for predicting small molecule–protein interactions relevant to MI biomarkers, including CK-MB, LDH, and cTn isoforms. Collectively, aegeline pronounced affinity and stabilizing effect on CK-MB, and to a lesser extent on LDH, cTnI, and cTnT, support its therapeutic candidacy for MI intervention, particularly via robust inhibitory action and stabilization of cardiac injury markers (67, 68).

Aegeline’s cardioprotective effects in ISO-induced MI may involve multitargeted interactions and pharmacological actions. After ISO administration, myocardial injury primarily occurs through oxidative stress, inflammation, and apoptosis, damaging biomarkers like CK-MB, LDH, and cardiac troponins. Aegeline, a natural alkaloid, binds strongly to CK-MB, stabilizing its function and reducing biomarker release and myocardial necrosis. It also boosts antioxidant defenses, lowers ROS, and suppresses inflammatory mediators like NF-*κ*B and TNF-α, preventing tissue damage. Moreover, aegeline inhibits caspase-3 activation, reducing cell death, and promotes conformational stability of cardiac proteins, supporting cardioprotection. It may improve cardiac contractility by modulating cTnT and cTnI and stabilizes membranes by regulating MDA. Overall, aegeline acts through antioxidant, anti-inflammatory, antiapoptotic, and protein stabilization mechanisms, making it a promising natural therapy for ISO-induced MI.

## Limitations

This research sheds light on the possible preventive benefits of aegeline against acute ISO-induced heart damage in rats. It investigated an acute model of heart damage, which provides useful insights but offers only a limited view of the disease process. To gain a more complete understanding, aegeline should be studied in a chronic model, such as coronary artery ligation, which exhibits long-term cardiac alterations. Additional hemodynamic indicators and signaling pathways, such as mitogen-activated protein kinase, may help us understand the mechanisms of heart injury and identify potential alternative therapeutic targets. A limitation of the present study is the absence of direct cardiac functional assessments. Although biochemical, inflammatory, oxidative stress, apoptotic, and histopathological parameters provided substantial evidence of cardioprotection, hemodynamic and echocardiographic measurements—such as left ventricular developed pressure, heart rate, ejection fraction, fractional shortening, and ventricular contractility—were not evaluated. These functional parameters would provide additional insight into the effects of aegeline on cardiac performance following myocardial injury. Future studies should incorporate both invasive and non-invasive cardiac function assessments to further establish the therapeutic potential of aegeline. The current mechanistic evidence is primarily based on ELISA measurements of inflammatory and apoptotic biomarkers. While these findings provide important insights into the cardioprotective effects of aegeline, additional validation of key signaling pathways, such as NF-*κ*B activation and caspase-3 cleavage, using direct molecular techniques (e.g., Western blotting or immunohistochemistry), would further strengthen the study’s mechanistic conclusions.

## Conclusion

The present study demonstrates the cardioprotective properties of aegeline against ISO-induced damage, as evidenced by amelioration of altered histopathological patterns, reduction in cardiac injury, improvement in antioxidant and oxidative stress markers, decrease in inflammatory markers, and inhibition of apoptotic expression. Importantly, there is no impact on liver and kidney function. This research suggests that aegeline may be a potential remedy for myocardial damage and identifies cTnT, TnI, MDA, TNF-α, NF-*κ*b, and caspase-3 as promising therapeutic targets.

## Data Availability

The original contributions presented in the study are included in the article/Supplementary Material, further inquiries can be directed to the corresponding author.
