# Supplementary material for: Aegeline mitigates isoproterenol-induced myocardial infarction: insights from biochemical, histopathological, and in silico studies
Source: Front Cardiovasc Med. 2026 Jul 21;13:1805096. doi: 10.3389/fcvm.2026.1805096 (PMC13433159; doi:10.3389/fcvm.2026.1805096)
Supplement: Supplementary file 1 [file Datasheet1.docx]

**Supplementary figure**

**Figure S1:** Ramchandran plot retrieved from the PROCKECK server.

**A	B**

**C	D**

**E	F**

**Figure S2A-F**: MD simulation analysis of 100 ns trajectories of RMSD of Cα backbone of **(A)** 3B6R_Aegeline **(B)** 7DBJ_Aegeline. RMSF of Cα backbone of **(C)** 3B6R_Aegeline **(D)** 7DBJ_Aegeline. Radius of Gyration (Rg) of Cα backbone of **(E)** 3B6R_Aegeline **(F)** 7DBJ_Aegeline

**A	B**

**C	D**

**Figure S3A-D**: MD simulation analysis of 100 ns trajectories of Formation of hydrogen bonds in **(A)** 3B6R_Aegeline **(B)** 7DBJ_Aegeline. MD simulation analysis of 1000 frames of the solvent accessible surface area of **(C)** 3B6R_Aegeline **(D)** 7DBJ_Aegeline**.**

**A	B**

**A	B**

**Figure S4A-D**: Bar graph of Protein-ligand contacts of **(A)** 3B6R_Aegeline **(B)** 7DBJ_Aegeline**.** Protein-ligand percent interactions of **(C)** 3B6R_Aegeline **(D)** 7DBJ_Aegeline**.**

**Figure S5:** Principal Component Analysis (PCA) plots of MD trajectories for the 3B6R–Aegeline (top panel) and 7DBJ–Aegeline (bottom panel) complexes. Scatter plots represent the distribution of conformations along the first principal components (PC1 vs PC2, PC2 vs PC3, and PC9 vs PC10). The percentage variance explained by each component is indicated on the axes. Frames are color-coded from red (early, 0 ns) to blue (late, 100 ns) to illustrate the conformational progression during the simulation.

**Figure S6:** Dynamic cross-correlation maps (DCCM) of MD trajectories for 3B6R–Aegeline (left) and 7DBJ–Aegeline (right) complexes. Blue regions indicate positively correlated motions, while red regions represent anti-correlated motions between residues. The maps highlight differences in global and local residue dynamics, with 3B6R showing more synchronized motions and 7DBJ displaying greater conformational flexibility.

**Figure S7:** Free energy landscape (2D and 3D) of the two protein–ligand complexes plotted against RMSD and radius of gyration. Blue basins represent the lowest Gibbs free energy states, indicating the most stable conformations, with the first complex showing a deeper, well-defined basin compared to the broader, scattered minima of the second complex.
